# Computerized Prediction of Radiological Observations Based on Quantitative Feature Analysis: Initial Experience in Liver Lesions

**DOI:** 10.1007/s10278-017-9987-0

**Published:** 2017-06-21

**Authors:** Imon Banerjee, Christopher F. Beaulieu, Daniel L. Rubin

**Affiliations:** Department of Radiology, Stanford University, Stanford, CA 94305 USA

**Keywords:** Visual semantic term, Annotation, Quantitative feature, Mapping, Liver lesion

## Abstract

We propose a computerized framework that, given a region of interest (ROI) circumscribing a lesion, not only predicts radiological observations related to the lesion characteristics with 83.2% average prediction accuracy but also derives explicit association between low-level imaging features and high-level semantic terms by exploiting their statistical correlation. Such direct association between semantic concepts and low-level imaging features can be leveraged to build a powerful annotation system for radiological images that not only allows the computer to infer the semantics from diverse medical images and run automatic reasoning for making diagnostic decision but also provides “human-interpretable explanation” of the system output to facilitate better end user understanding of computer-based diagnostic decisions. The core component of our framework is a radiological observation detection algorithm that maximizes the low-level imaging feature relevancy for each high-level semantic term. On a liver lesion CT dataset, we have implemented our framework by incorporating a large set of state-of-the-art low-level imaging features. Additionally, we included a novel feature that quantifies lesion(s) present within the liver that have a similar appearance as the primary lesion identified by the radiologist. Our framework achieved a high prediction accuracy (83.2%), and the derived association between semantic concepts and imaging features closely correlates with human expectation. The framework has been only tested on liver lesion CT images, but it is capable of being applied to other imaging domains.

## Background

Next-generation medical informatics approaches aim to go beyond analyzing radiological images (the basis of most current computerized diagnosis systems), to integrating the image data and their semantics, providing a single, unified, and searchable data structure. Such integrated data could support automated reasoning on both image content and image semantic levels to better infer diagnoses and inform management decisions. However, since radiologists record their imaging observations in free text reports, the “semantic content” of the image data is not easily machine accessible. Moreover, inter-observer variability and inappropriate/incomplete imaging observations limit their usefulness in the clinical environment [1].

Computer-assisted image annotation with controlled vocabulary could overcome the aforementioned limitations, but granular-structured reporting challenges efficient radiology workflow, and automated semantic annotation of radiological images is still a challenging research direction. We divide the semantic annotation problem into two major subproblems: (i) extracting quantitative imaging features (“low-level features”) and (ii) reducing the “gap” between low-level imaging features and the imaging observations reported by radiologists, hereafter called “visual semantic terms” (VSTs). The VSTs are often standardized terms—ideally from controlled terminologies—that the radiologists use in their reports, such as clinical indications, anatomy, visual features of abnormalities, diagnostic interpretations, and recommended management [2]. Recognition of VST patterns can have a strong influence on the final diagnostic decision made by radiologists, since many diagnoses are characterized by particular VSTs or combinations of them.

Much of the recent focus in computer vision has been on finding the solution for the first subproblem, and there are currently many methods that can compute a wide range of quantitative parameters from 2D/3D images to represent quantitative aspects of image contents [3]. However, the second subproblem remains challenging because a good feature is not always the *best feature* for representing a VST, and, yet, there is no clear association between the VSTs and the low-level image features. A number of studies [4, 5] have been undertaken that create computerized models for predicting VSTs from the raw image by computing a fixed set of low-level image features and solving a classification problem. However, the classification decisions taken by such models can be difficult for a radiologist to reason about since the models either derive a binary output or assign a probability or confidence score. Without a formalized mechanism to reason about why an automatic classifier detects the set of VSTs, clinicians tend to distrust them. On the other hand, the *low-level imaging features lack semantic meaning*, and therefore, the classification decisions made by a combination of low-level features are difficult to justify to radiologists and clinicians, who typically use subjective heuristics to diagnose patient cases.

A precise mapping between the low-level quantitative image features and the high-level VSTs can help radiologists better understand computerized prediction results, and the mapping can even incorporate the expert feedback on the prediction results. In addition, mappings between VSTs and quantitative imaging features could be leveraged to infer additional semantic concepts that were not even considered before for the targeted prediction and could improve radiological image interpretation.

We propose a framework that not only predicts radiological semantic terms from low-level quantitative image features but also derives explicit association between the low-level imaging features and VSTs based on statistical correlation. We make two key research contributions. *First*, we develop a VST detection algorithm called “An iterative approach for Partial Max Dependency with Equal Importance” (PmEI) that maximizes the feature relevancy for each targeted VST while giving a fair chance to all low-level quantitative imaging features to be included in the learning. The main hypothesis behind *PmEI* scheme is *if the correlated features are demonstrating nearly equal relevancy with the targeted annotation, they deserve to be incorporated in the machine learning phase with equal importance*. In the PmEI algorithm setting, we test the significance of a wide range of low-level imaging features targeting the liver lesion CT image annotation task. As a *second contribution*, we design a specialized feature that is computed in an automatic way by analyzing CT image of the liver to quantify lesion(s) present within the liver which have similar appearance as the primary lesion identified by the radiologist.

For this initial analysis, we implemented our framework as a prototype that analyzes liver lesion CT images and that achieved 83.2% overall VST prediction accuracy. The remaining article is organized as follows: Second section describes our methodology, third section describes experimental results, and fourth section presents a summary of the work and some concluding remarks.

## Methods

**Fig. 1:**
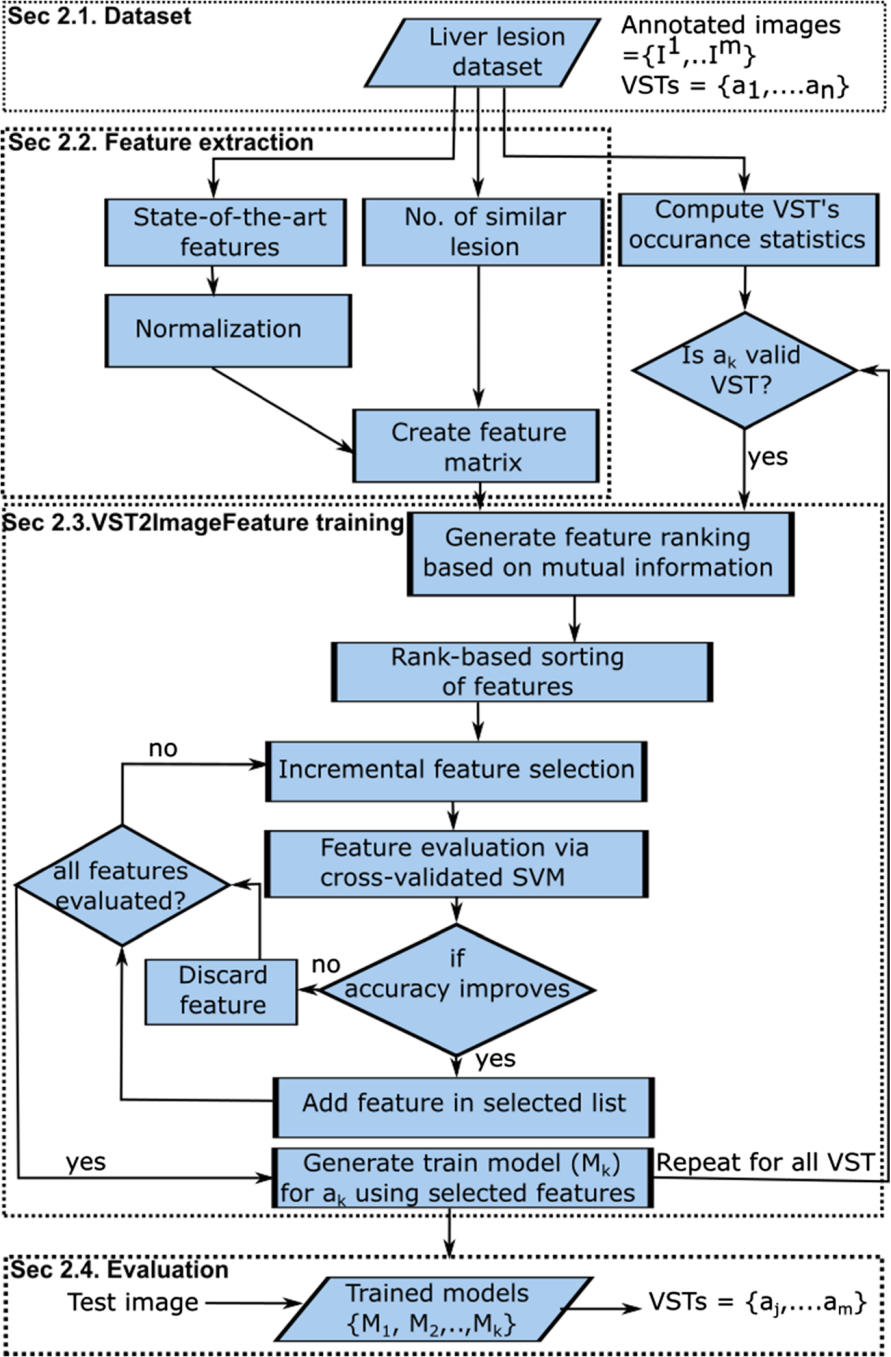
Workflow for predicting clinical imaging findings

Figure 1 presents a summary of the proposed workflow for VST prediction. A set of expert-annotated CT liver lesion images are used to train and validate the system (see “Dataset: Annotated Liver Lesion in CT Images” for details). The *feature extraction* block computes a wide variety of traditional and a novel quantitative feature from each image by considering the lesion’s intensity and texture characteristics and its shape and position, and it concatenates all the individual feature vectors to create a 496-dimensional feature matrix. The dimension of individual traditional features (495 dimensions) is mentioned in Table 1, and we also compute a novel quantitative feature that captures the number of similar appearing lesions within the liver. The *VST2ImageFeature* training block iteratively learns the mapping between the 496 different quantitative features that represent lesion characteristics at the pixel level and 21 valid VSTs by using incremental search in the image feature space employing the PmEI algorithm. By employing the pre-trained model, the *prediction* block can automatically annotate unseen CT liver images with a set of pre-defined VSTs. We detail each core block (dotted in Fig. 1) in the following sections, and corresponding section indices are also mentioned within the figure. We present the results in “Results” section.

**Table 1:** Quantitative features used in this study (literature citation and acronym are mentioned where necessary)

Type	Name and citation	Dimension	Represents
Intensity-based features	Intensity median inside lesion—IntensityM	1	Quantify 1st order intensity distribution within the lesion
	Entropy inside lesion—Entropy	1	
	Proportion of pixels with intensity larger than pre-defined threshold—ProportionThres	1	
	Intensity different between lesion and its neighboring tissue (3 scale analysis)—IntensityDiff	3	
Texture features	Haralick features—GLCM ([6]	12	Capture occurrence of gray level pattern within the lesion.
	Gabor features—Gabor [7]	32	
	Daubechies features—Daube [8]	324	
	Haar wavelets—Haar	1	
	Run Length Matrix—RLE [9]	7	
Shape features	Compactness [10]	1	Describe the morphology of the lesion
	Eccentricity	1	
	Roughness [11]	1	
	Local area integral invariant—LocalIntegral [12]	15	
	Radial distance signatures—RadialSig [13]	2	
Histogram-based feature	Local binary pattern—LBP [14]	12	Compute marginal distribution of gray values with in lesion
	No. of pixels in different hist. bins—Histogram-bin	20	
Edge-based features	Edge sharpness	60	Quantify edge sharpness along the lesion contour
	Histogram on edge—EdgeHist	1	

### Dataset: Annotated Liver Lesion in CT Images

**Fig. 2:**
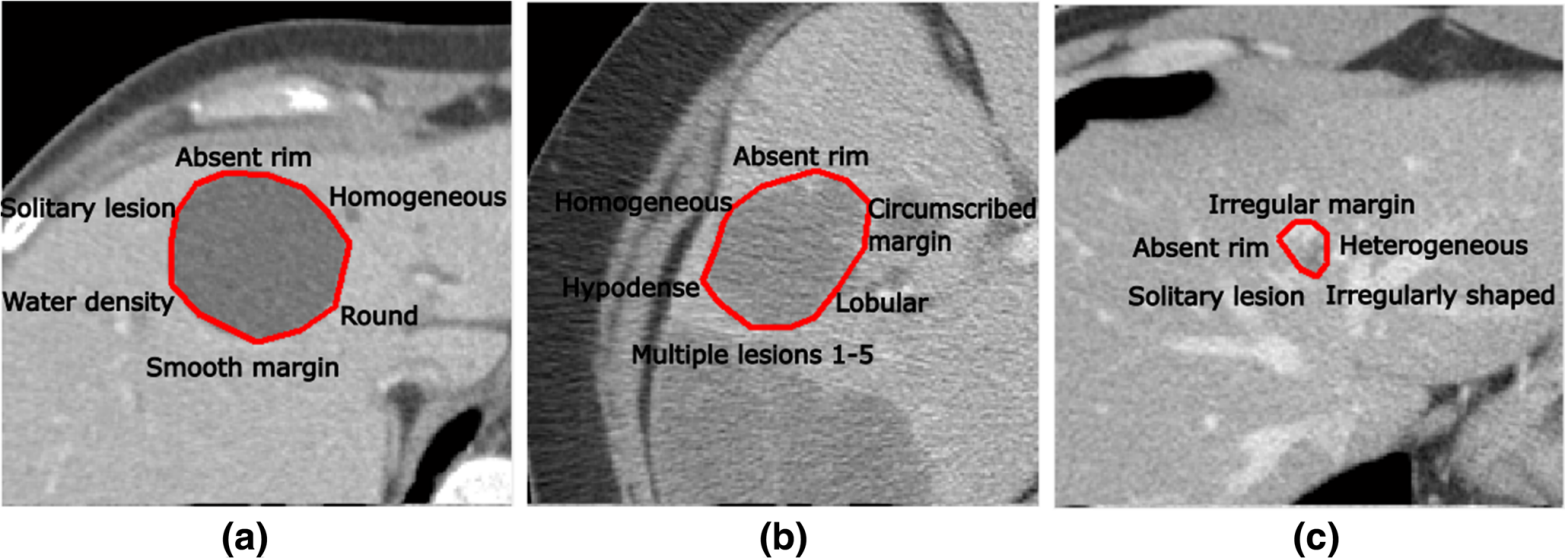
Samples from liver lesion dataset. **a** Cyst. **b** Metastasis. **c** Hemangioma. The boundaries of the lesions are highlighted in *red*, and a subset of annotated VSTs are written in *black*

With the approval of the institutional review board (IRB), we used a dataset containing 79 contrast enhanced de-identified CT images of patients having liver lesions, obtained in the portal venous phase with a slice thickness of 5 mm [4]. The dataset contains three common types of liver lesions: cyst, metastasis, and hemangioma (see Fig. 2). On each scan, a radiologist (with more than 16 years’ experience) annotated the ROI circumscribing each liver lesion in a mid-axial slice that contains the largest lesion area using Electronic Physician’s Annotation Device (ePAD) [15]. The RadLex ontology [16] was used to define most of the *VST*s for image annotation; a few additional descriptive terms not in RadLex were also used to describe the liver lesions and thus included in the VST set used for image annotation.

**Fig. 3:**
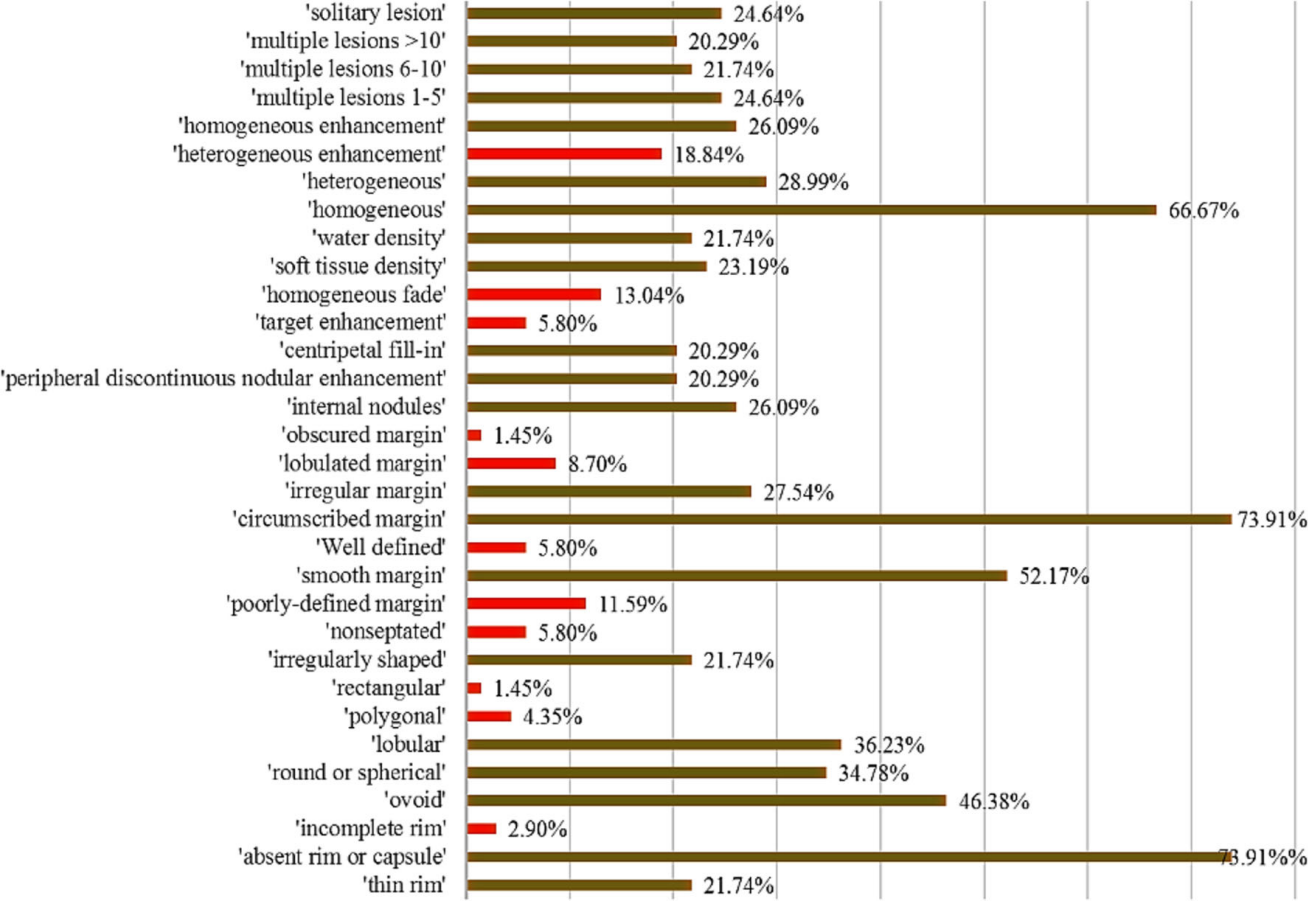
VST occurrence statistics in the liver lesion CT dataset

Each lesion was annotated with 10–12 VSTs, and the final diagnosis was recorded. All VSTs were not equally used throughout the dataset, since the number of samples is not equally distributed among each diagnosis class and even lesions with same diagnosis had a varying visual appearance, resulting in different annotations. Therefore, we compute the statistics of VST occurrences within the dataset, and in Fig. 3, we present the outcome. To avoid overfitting in the learning stage, we consider only the VSTs that have occurrence between 20 and 75% (i.e., the green bars in Fig. 3). Given this restriction, in this study, we created annotation models for 21 valid VSTs.

### Feature Extraction Block

As seen in Fig. 1, we extracted a set of quantitative features that includes state-of-the-art quantitative features from the primary lesion and its neighboring healthy tissue and a new feature that represents the number of similar appearing lesions within the liver. Finally, we concatenate them to create a single feature matrix.

#### State-of-the-art Quantitative Features

In Table 1, we list quantitative features that we computed. In total, we incorporated features from five distinct categories and the overall dimension of our feature matrix is 495. We compute the features in their standard configuration, and afterwards, we normalized the feature vector to have 0 mean and 1 standard deviation.

#### A New Feature: “Number of Similar Lesions”

**Fig. 4:**
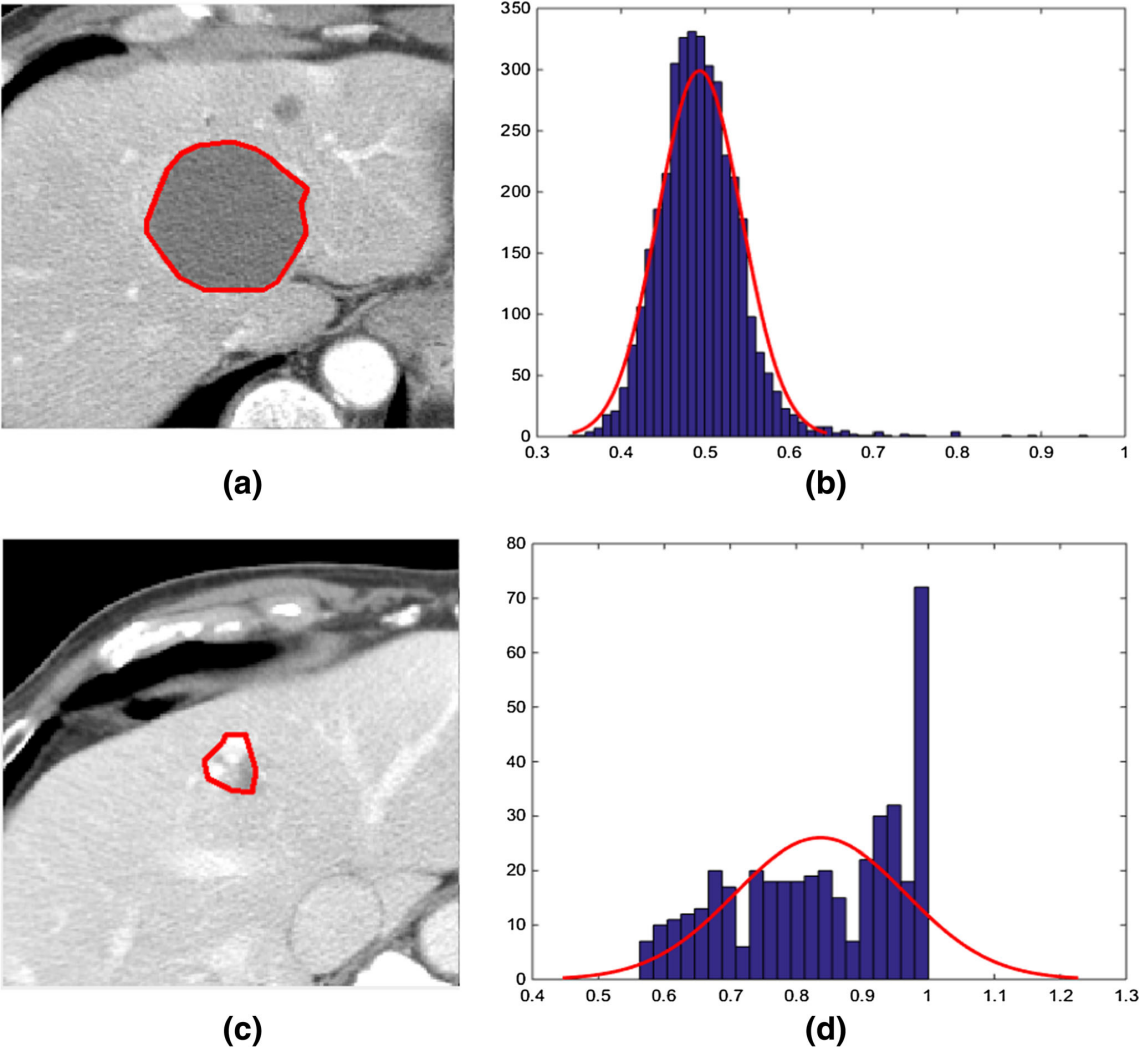
Primary liver lesions identified by the radiologists: a homogeneous lesion (**a**) where the pixel values within the lesion follow normal distribution (**b**) and a heterogeneous lesion (**c**) where the pixel values within the lesion do not follow normal distribution (**d**)

We established a VST, the “lesion load” that represents the count of similar appearing lesions within the liver, since this is one of the frequently appearing imaging observations for lesions in the liver. To create this VST, we constructed a pipeline that automatically recognizes and counts the lesions that have an appearance similar to the primary lesion. First, we apply a within class variance and intensity-based thresholding method [17] to recognize the potential candidate pixels for the lesion within a cropped version of the original CT image that represent the region inside the liver and its surrounding region. The thresholding criterion is defined in Eq. (1).

1 $$ J\left(\lambda, T\right)=\left(1-\lambda \right){\sigma}_{\mathrm{w}}(T)-\lambda \mid {m}_{\mathrm{l}}(T)-{m}_{\mathrm{b}}(T)\mid $$

where *m*_l_(*T*) and *m*_b_(*T*) are, respectively, the mean intensity of primary lesion and the background, and *σ*_w_ is the square root of within class variance, defined as:

2 $$ {\sigma_{\mathrm{w}}}^2={P}_{\mathrm{l}}(T){\sigma}_{\mathrm{l}}^2+{P}_{\mathrm{b}}(T){\sigma}_{\mathrm{b}}^2 $$

where *P*_l_(*T*) *σ*_l_ and *P*_b_(*T*) *σ*_b_ are, respectively, the probabilities and corresponding variances of the lesion and the background. In Eq. (1), *λ* is a weight which determines the balance between intensity profile and within class variance and the value is ranged between 0 and 1. The value of *λ* has been chosen as 0.75, giving more weights to the lesion intensity profile for homogeneous lesion primary lesion where 68% pixel of the primary lesion area falls within the range of ±*σ*_l_ (normal distribution) (Fig. 4a, b). For highly heterogeneous primary lesion where the distribution of pixel value does not follow the normal distribution (Fig. 4c, d), the value of *λ* has been chosen as 0.15, giving more weights to the within class variance. Afterwards, the optimum threshold *T* ^∗^ is selected by optimizing Eq. (3).

3 $$ J\left(\lambda, {T}^{\ast}\right)=\underset{T}{ \min}\left( J\left(\lambda, T\right)\right) $$

**Fig. 5:**
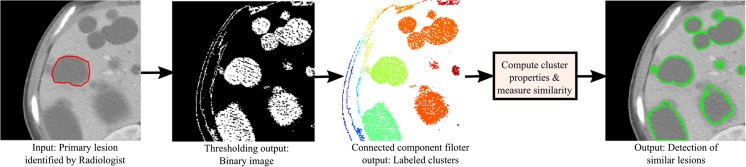
Automatic similar lesion identification pipeline in CT liver image

The pixels identified by the thresholding process are not contiguous (see Fig. 5 thresholding output). Hence, we apply the flood-fill algorithm to identify the connected components from the binary image generated by thresholding and label them accordingly. We analyze the labeled image where the candidate pixels are grouped in separated clusters and evaluate two intrinsic properties of each cluster: (1) 1st order statistical property (*α*)—difference in mean and standard deviation of the pixels exist inside and two ring neighbors outside cluster and (2) shape property (*β*): the shape of cluster represented by a ratio of major and minor axis lengths. To measure the similarity, we compute the Euclidean distance between the primary lesion (*p*) and the new candidate cluster (*c*_i_) in a space built by considering the *α* and *β* as in Eq. (4).

4 $$ d\left( p,{c}_{\mathrm{i}}\right)=\sqrt{{\left({\alpha}_p - {\alpha}_{c_{\mathrm{i}}}\right)}^2+{\left({\beta}_p - {\beta}_{c_{\mathrm{i}}}\right)}^2} $$

If the distance between primary lesion and candidate cluster (*d*(*p*, *c*_i_)) is less than or equal to an empirically defined threshold value, we label the cluster as “identical lesion” or otherwise discard the cluster.

In Fig. 5, we show the complete pipeline as well as step-by-step results achieved from a single sample image. We utilize the final output to quantify the number of similar lesions which we then incorporate as a single scalar value feature in the feature matrix: “number of similar lesion.”

### VST2ImageFeature Training: an Iterative Learning Based on PmEI

The VST2ImageFeature training block (see Fig. 1) establishes an association between the quantitative image features (i.e., automatically computed from the images) and the VSTs (i.e., the annotations done by radiologist) and finally utilizes the selected features to predict the VSTs. We formulated an iterative approach for learning a mapping between low-level image features and VSTs that maximizes the feature relevancy for each targeted VST (*a*_*k*_). We named it *partial max-dependency* algorithm since the algorithm considers only the *max-relevancy* criteria of the *max-dependency algorithm* [18] and ignores the *min-redundancy* part of it. We provide equal chance to all the features to be included in VST learning, even if the low-level features have correlation among themselves. The intuition behind this method is *if the correlated features exhibit nearly equal relevancy with the targeted annotation, they should be incorporated in the learning phase with equal importance*. But often, inclusion of highly correlated features does not change the class-discriminative power. Thus, we aim to increase the accuracy of the model for predicting the VSTs by recursively evaluating the classification error for each incremental *feature selection* step (see Fig. 1).

- Step 1: In our liver lesion dataset, VSTs are not mutually exclusive, thus multi-class classification cannot be formulated directly. We formulated a binary classification problem by representing each VST_*k*_ as targeted annotation variable: *a*_*k*_ ∈ {1, −1}, where *a*_*k*_ = 1 if the VST_*k*_ is present or else *a*_*k*_ =  − 1. For the liver lesion dataset, we consider total 21 VSTs, and therefore, we have the annotation set *a* as: *a* = {*a*_1_ , *a*_2_ , … . ., *a*_*m*_ } , where *m* = 21.
- Step 2: The algorithm follows a partial *max-dependency* approach [18] where the features *Q*Features = {*f*_1_, *f*_2_, … . ., *f*_*n*_ } are first ordered according to their relevancy (*R*) to the targeted annotation (*a*_*k*_). The relevancy is characterized by the mutual information [19] between the individual feature (*f*_*j*_) and the targeted annotation (*a*_*k*_). Mutual information (mutualInfo(*f*_*j*_, *a*_*k*_)) measures how much knowing the feature variable (*f*_*j*_) reduces uncertainty about the annotation (*a*_*k*_). Formally, mutual information between feature *f*_*j*_ and annotation *a*_*k*_ can be computed as:

5 $$ \mathrm{mutualInfo}\left({f}_j,{a}_k\right)= H\left({f}_j,{a}_k\ \right)- H\left({f}_j|{a}_k\right)- H\left({a}_k|{f}_j\right) $$

where *H*(*f*_*j*_, *a*_*k*_ ) is joint entropy and *H*(*f*_*j*_| *a*_*k*_) , *H*(*a*_*k*_| *f*_*j*_) are conditional entropy [20]. Accordingly, the max feature relevancy for an annotation *a*_*k*_ is defined as:

6 $$ {R}_{\max}\left({a}_k\right)={ \max}_{j=1}^n R\left({f}_j,{a}_k\right),\kern1em \mathrm{and}\  R\left({f}_j,{a}_k\right)=\mathrm{mutualIn} fo\left({f}_j,{a}_k\right) $$

where *n* = no. of quantitative features. Afterwards, we create a sorted the feature matrix *F* = {*f*_*j*_, *f*_*j* + 1_, … . .}_*n*_, where *R*(*f*_*j*_, *a*_*k*_) ≥ *R*(*f*_*j* + 1_, *a*_*k*_).

**Figure 6:**
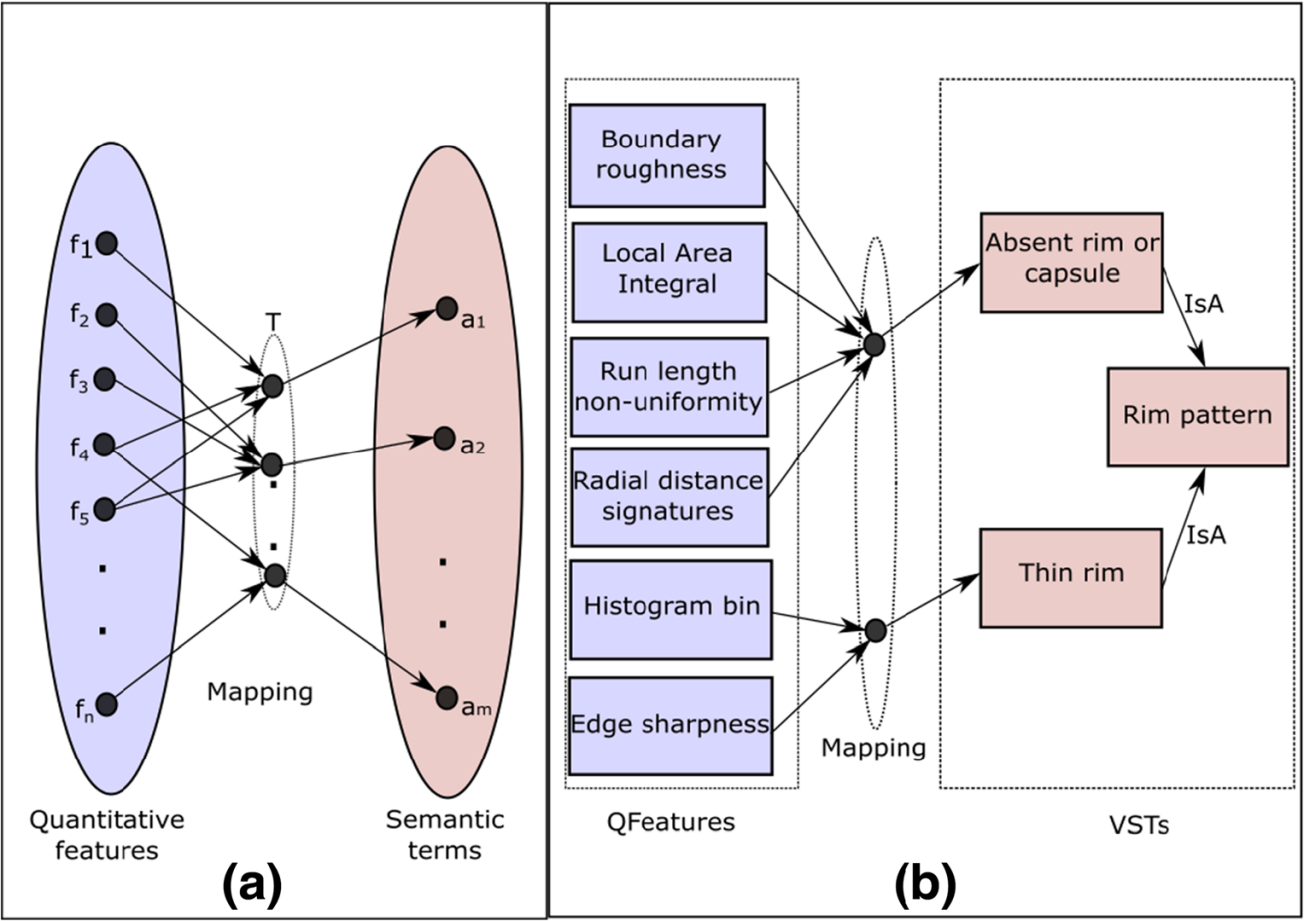
Mapping between quantitative features and visual semantic terms: **a** conceptual representation and **b** derived mapping for lesion rim pattern VSTs.

- Step 3: To minimize the classification error with *an optimal set of candidate feature set*, we follow an iterative learning approach where incremental selection of the features from the sorted feature matrix *F* is performed if and only if including the current feature leads to better a classification accuracy. We start by initializing a selected feature list (*SF*) by considering the first feature from the sorted feature matrix *F*, such that SF = {*f*_1_ }, where *R*(*f*_1_, *a*_*k*_) ≥ *R*(*f*, *a*_*k*_) ,  ∀ *f* ∈ *F*. Now, on the SF feature space, we train a non-linear *support vector machine* (SVM) [21] model with Gaussian kernel for classifying the particular VST (*a*_*k*_).
  To reduce the overfitting in the training, we use fivefold cross-validation SVM: we first divide the training set (79 samples) into five subsets of equal size using random sampling and one subset is tested using the classifier trained on the remaining (5–1) subsets. The cross-validation accuracy metrics is defined as the portion of data which are correctly classified: $\tau =\frac{\mathrm{true}\ \mathrm{positive}+\mathrm{true}\ \mathrm{negative}}{\mathrm{Total}\ \mathrm{no}.\kern0.5em \mathrm{of}\ \mathrm{sample}}$. For the first iteration, we represent the cross-validation prediction accuracy as *τ*_1_.
- Step 4: Afterwards, in each step (*i*), we apply a forward-search strategy which iteratively considers the next feature (*f*_*i*_) from the remaining feature list (*F*_(*n* − *i*)_) that has the maximum relevancy value with the targeted annotation (*a*_*k*_), and we form a temporary feature list as: =SF∪ *f*_*i*_. We execute *fivefold cross-validated SVM* considering the space formulated by temporary feature list (Temp*F*). If the cross-validation prediction accuracy of the current learning (*τ*_*i*_) is greater than the previous accuracy (*τ*_(*i* − 1)_), we include the new feature in the selected list (i.e., SF = Temp*F*) or else discard the feature, and iterate step 3 with next feature from the remaining feature list (*F*_(*n* − (*i* + 1))_). We execute steps 2–4 for each annotation presents in the annotation set *a*. Note that the number of selected features for each annotation *a*_*k*_ are chosen dynamically by accuracy evaluation.
- Step 5: After iterating over the steps 2–4 for each VST (i.e., we iterated for 21 times for the current dataset), the algorithm derives an optimal mapping between subsets of quantitative imaging features and the VSTs. The mapping *T* is defined as: *T* : Sub*Q*Features → *a*_*k*_ where Sub*Q*Features ⊆ *Q*Features and *a*_*k*_ ∈ *a* (Fig. 6a). For giving an example, in Fig. 6b, we present the derived mapping for two types of *rim patterns*—“absent rim or capsule” and “thin rim.”

### Prototype Development and Evaluation

**Fig. 7:**
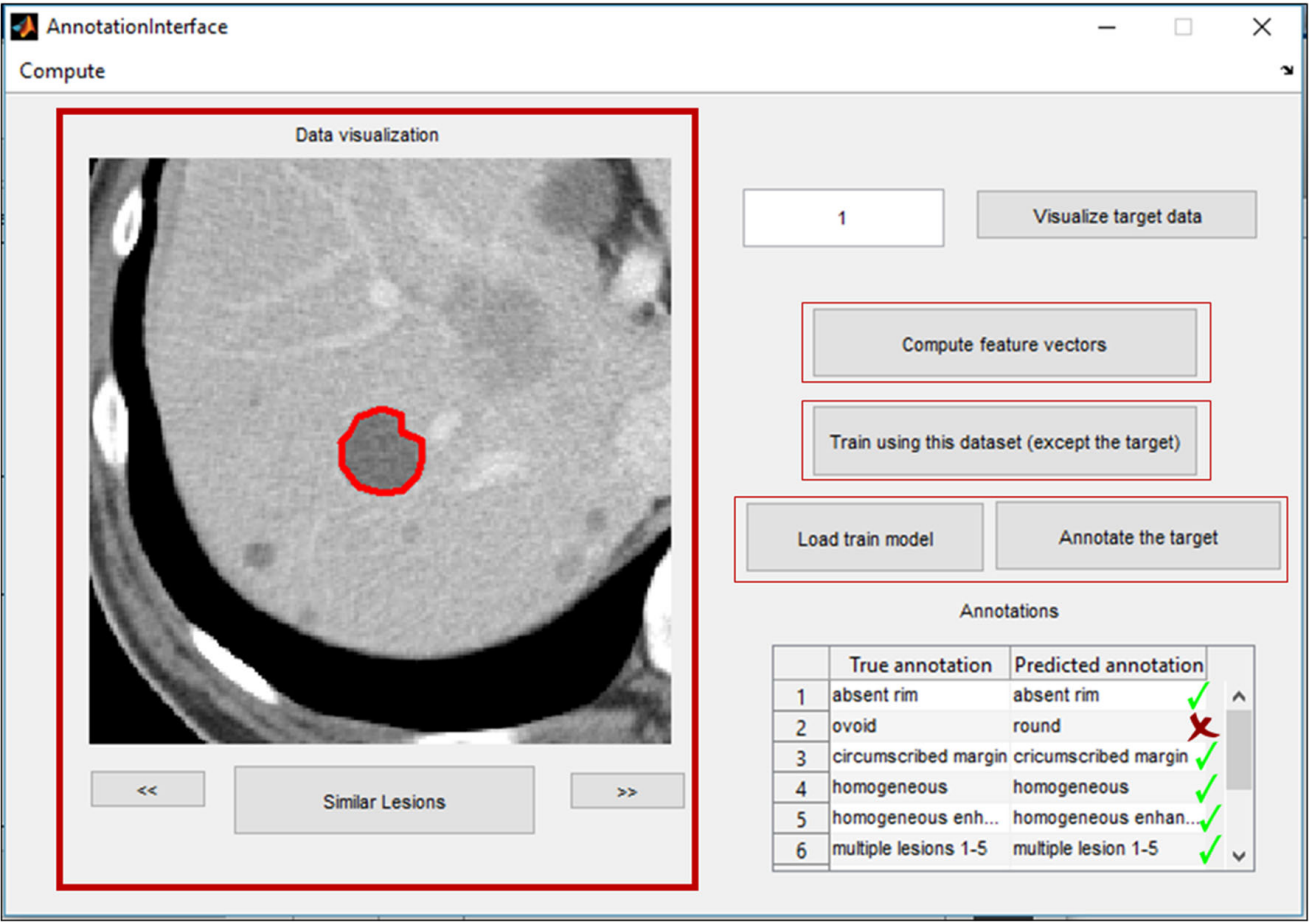
MATLAB GUI snapshot showing automatic semantic annotation results for a sample data

We implemented the various components of the proposed workflow (Fig. 1) and developed a MATLAB application that allows user to load the CT image and the user-defined ROI, compute quantitative features, derive the mapping of quantitative features to VSTs, and create a new trained model. Afterwards, with a single mouse click, the trained model can be exploited to perform completely automatic VST identification for unseen CT liver lesions. In Fig. 7, we present a snapshot of our application that shows the automatic annotation result for a CT sample image, where the “true annotation” and the “predicted annotation” columns show the annotations done by the radiologist and the annotations predicted by our system.

We evaluated our MATLAB prototype on the liver lesion CT dataset of 79 images. A primary lesion in each image/sample was identified and annotated by an experienced radiologist using a controlled vocabulary of 76 VSTs (Fig. 3) [16]. As mentioned earlier (“Methods” section), different classes of liver lesions are included in our dataset and they demonstrate a variety of visual appearance in CT images. Therefore, the VST occurrence also varied widely among the samples (see Fig. 3). In total, we created a model to predict the presence or absence of 21 unique VSTs by considering 496 different quantitative features. The core functionalities of our system were evaluated in two ways. First, we compared the fivefold cross-validation VST prediction accuracy with the ground truth created by the radiologist (recorded VSTs). Second, we performed a 2-stage evaluation where expert radiologists analyzed the feature-to-concept mapping outcome, considering the expert expectation of correlation (individual belief). In the following section, we summarize the results of the evaluations.

## Results

### VST Prediction Accuracy

**Fig. 8:**
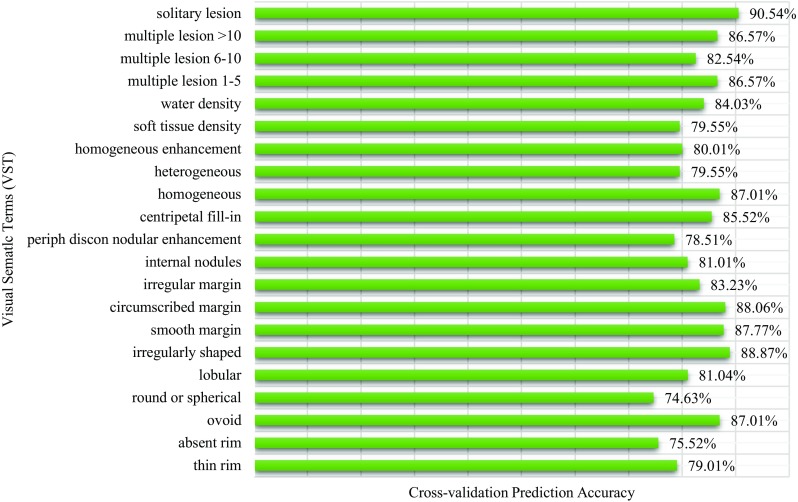
Individual VST cross-validation prediction accuracy measured by executing PmEI method

In Fig. 7, we present an automatic annotation result for an unseen liver lesion sample. For most VSTs in this example, the predicted annotations match with the manually defined ground truth. Only the “ovoid” was predicted wrongly as “round.” For this lesion, this error is very likely since both ovoid and round can be applicable for describing the lesion shape as the shape is neither perfectly round nor absolutely ovoid/egg-shaped yet possesses both characteristics. Figure 8 presents the statistics of fivefold cross-validation prediction accuracy for all 21 VSTs where the original dataset is randomly partitioned into five subgroups, one subgroup is left out in each iteration of training, and finally, the unseen subgroup is used to test the model’s performance. The highest accuracy achieved is 90.54% for the VST “solitary lesion,” the lowest is 74.63% for the VST “round or spherical,” and the average accuracy for 21 VSTs is 83.2%. For some VSTs, a possible reason behind getting relatively low accuracy is that either the terms do not have any clear independence from another VST, e.g., ovoid vs round or spherical, or the statistic of occurrence of the VST is not properly balanced, e.g., absent rim.

### Mapping Between Quantitative Imaging Feature and Visual Semantic Terms

**Fig. 9:**
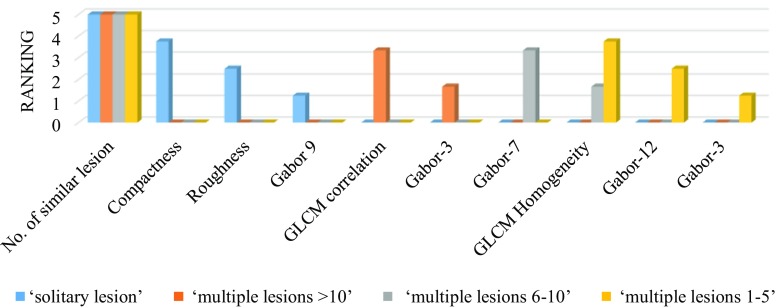
PmEf feature selection outcome for VSTs representing liver lesion load

**Fig. 10:**
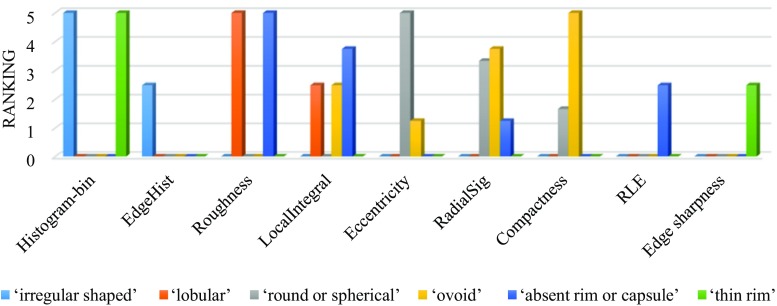
PmEf feature selection outcome for VSTs representing lesion shapes and rim patterns

**Fig. 11:**
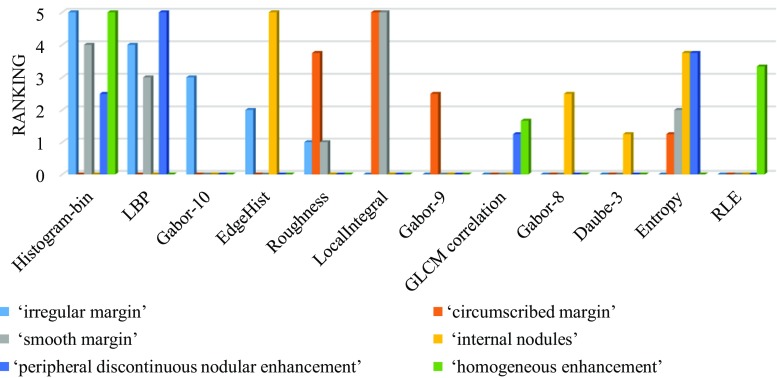
PmEf feature selection outcome for VSTs representing lesion margins, presence of nodule, and enhancement pattern

**Fig. 12:**
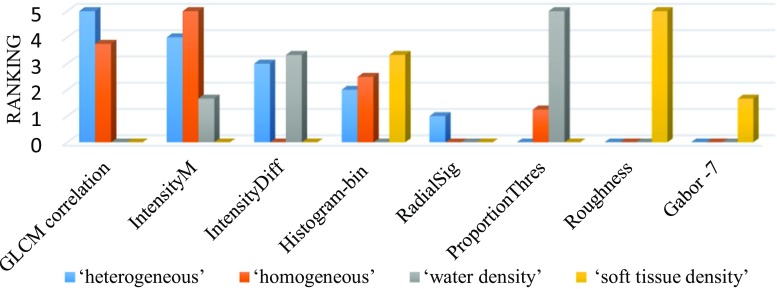
PmEf feature selection outcome for VSTs representing uniformity descriptor and density related entity

From the large quantitative feature matrix of dimension 496, our iterative learning algorithm (*PmEf*) automatically creates the mapping between the VSTs and the discriminative features by considering two primary constrains: (i) significant dependency with the targeted VST and (ii) improved cross-validation prediction accuracy when it is being incorporated within the learning model. Figures 9, 10, 11, and 12 present the feature mapping results for the set of VSTs considered in our current study where the colored bars represent VSTs, the vertical axis represents the ranking value, and the horizontal axis represents the quantitative image features. We represent the ranking value in the scale of 0–5 where 5 represents the most informative feature and 0 means that the feature is not mapped with the VST. A validation of the feature to concept mapping from a clinical perspective can be very challenging since the low-level quantitative features lacks the semantics and therefore cannot be directly interpreted by the humans.

We performed a 2-stage evaluation. We first analyzed the mapping from a computer vision perspective and, afterwards, conducted individual sessions with two expert radiologists specialized in abdominal imaging where we compared computer-derived mapping with the expectation in an abstract level. We observed that inter-radiologist agreement was very high which makes comparison task straightforward. In the remaining section, we present a condensed version of what we derived from the 2-stage evaluation.

On average, our method mapped a single VST to only 4 quantitative features among 496 feature vectors compared with a prior method [4] that mapped each VST with 12.6 features for the liver lesion CT images. After analyzing the derived mapping from a computer vision perspective, we found that in most cases the automatically mapped quantitative features by our algorithm resemble the human expectation of the correlation. For instance, the VSTs related to lesion multiplicity (see Fig. 9) are mapped with the number of similar lesions (see “Methods” section), which quantifies similar appearing lesion(s) within the liver and the second order statistical features (GLCM, Gabor) and that represent spatial repetition of gray value arrangement within the liver. In contrast, the solitary lesion is mapped with the lesion shape and boundary features.

The VSTs that define the primary lesion shape (Fig. 10) are mapped mostly with the quantitative features that represent the 2D shape of the contour, identified by the radiologist. For instance, “eccentricity” and “RadialSig” features are the dominant for representing round or spherical lesion shape and “compactness” feature which represents the degree to which a shape is compact is derived as the most dominant for representing ovoid lesion shape. Further, the VSTs related to the rim pattern (Fig. 10) are associated with the imaging features that mainly describe the boundary characteristics of the 2D contour, e.g., “LocalIntegral” and “edge sharpness,” which is again highly analogous to the expectation.

Furthermore, local area integral invariant which computes integrals on the boundary and represents the local characteristics of the lesion boundary is derived as the most informative feature for both smooth and circumscribed lesion margin (Fig. 11). In contrast, the histogram and texture features are ranked higher for predicting the “irregular margin” (Fig. 11) which is reasonable since the margin irregularity is not trivial to be described by the lesion boundary characterization.

GLCM correlation and intensity-based features mapped consistently with both “heterogeneity” and “homogeneity” uniformity descriptors (Fig. 12). Proportion of pixels with intensity larger than pre-defined threshold value is the most dominant predictor for “water density,” and “roughness” is most dominant for “soft tissue density.” Interestingly, the texture features, such as “Gabor” and “histogram”, are mapped with soft tissue density whereas intensity-based features, such as intensity mean inside lesion and intensity difference with the neighborhood, are mapped with water density (Fig. 12).

## Discussion

Computerized radiological image interpretation is being widely studied. In prior work, creation of links between semantic terms and quantitative image features was mainly explored in two parallel ways: (i) bag of visual words approach (BoVW) and (ii) direct modeling of VSTs. Following the BoVW approach, André et al. [22] applied Fisher-based method for transforming visual word histograms learned from scale-invariant feature transform (SIFT) into eight visual semantic terms, but the transparency of the algorithm in terms of understanding is limited. Liu et al. [23] developed a bag of semantic word model that used a supervised sparse autoencoder to derive disease class patterns from neuro-imaging datasets. However, the learned patterns were not associated with any formalized semantic terms, and, therefore, the interpretation of the patterns is limited. Often, the core limitation of the BoVW-based approaches is that the visual words are not semantically meaningful, and there is a need to find the appropriate quantization granularity to find the mapping with semantic terms. This limits the effectiveness and compactness of the representation.

Moving towards the direction of direct VST modeling, Barb et al. [24] developed a computational mechanism for associating intensity-based image features and VSTs which also considers the perspective of individual users. Raicu et al. [25] developed a probabilistic model to predict lung nodule semantics using a set of quantitative image features (shape, size, gray-level intensity, and texture). Gimenez et al. [4] computed a relatively large set of quantitative features from liver CT images and fed the whole feature matrix in LASSO regularization model to predict the presence of VSTs. Depeursinge et al. [5] adopted a different modeling approach and created a SVM model using only the rotation-covariant Riesz wavelet features to learn the signature of each VST from liver CT images. To the best of our knowledge, up to now, no study was performed to build a quantitative model that can derive a human-interpretable mapping between the low-level image features and the high-level VSTs.

Our proposed framework not only predicts radiological VSTs but also derives explicit mapping between low-level imaging features and high-level VSTs based on statistical correlation. Therefore, the proposed system would be expected to be more intuitive and, perhaps, trustworthy, to human experts as they provide feedback on the prediction outcome. We have experimented with a large group of popular state-of-the-art quantitative features and also proposed a novel feature that can represent lesion multiplicity. We adopted an iterative-learning approach for the mapping which maximizes the feature relevancy for each targeted VST while giving a fair chance to all the features to participate in the learning. First, we extract a wide range of features from the CT liver lesion dataset and order the features as per their relevancy to the targeted VST. Second, the features are added iteratively using a subset evaluation strategy which ensures good performance for VST prediction. Our results (see “VST Prediction Accuracy” section) appear better than the average accuracy reported in a prior method [4] applied to a similar dataset.

The main limitation of our algorithm is that it follows an incremental greedy strategy for feature mapping: Once a feature has been selected, it cannot be deselected at a later stage. We plan to overcome this limitation by incorporating a hybrid search mechanism and evaluate the model on a large and balanced dataset. Also, a large part of evaluation adopted in this study is qualitative and validated by two radiologist experts in abdominal imaging (see “Mapping Between Quantitative Imaging Feature and Visual Semantic Terms” section) since it is not feasible to quantitatively measure legitimacy of the mapping between low-level quantitative and high-level semantic features. We plan to involve more experts from different domains and do a more intensive evaluation.

## Conclusion

We propose a system that automatically learns the mapping between low-level image features and high-level semantic concepts given a valid training dataset, and detect lesion characteristics from liver CT images by only given the lesion outline. The current framework has been experimented on a CT image dataset which contains three different types of liver lesion: cyst, metastasis, and hemangioma, but it can be easily adapted to a different annotation task. For instance, the system might also be able to predict the radiological observations from suspected a lesion area (non-human analyzable)—provided there are sufficient discriminating underlying machine-observable features—which may help in early-stage treatment planning. The automatically derived feature-concept mapping can improve the expressive power of computer-assisted radiological image annotation and can be leveraged to build a powerful tool for extracting “human interpretable explanation” from computer-aided diagnosis systems. Further, the mapping can be exploited to reduce the “semantic gap” between the user’s conceptualization of a textual query for retrieving radiological images and the low-level query that actually specify the image characteristics. This direction has the potential to enhance the efficiency of radiological image retrieval and browsing systems.
